# Thermal Annealing Effects of V_2_O_5_ Thin Film as an Ionic Storage Layer for Electrochromic Application

**DOI:** 10.3390/ma15134598

**Published:** 2022-06-30

**Authors:** Tien-Chai Lin, Bai-Jhong Jheng, Hui-Min Yen, Wen-Chang Huang

**Affiliations:** 1 Department of Electrical Engineering, Kun Shan University, Yongkang, Tainan 710303, Taiwan; tienchai@mail.ksu.edu.tw (T.-C.L.); chris81809@gmail.com (B.-J.J.); 2 Green Energy Technology Research Center, Kun Shan University, Yongkang, Tainan 710303, Taiwan; ec9526@mail.ksu.edu.tw

**Keywords:** electrochromic, ionic storage layer, V_2_O_5_ thin film, RF magnetron sputtering

## Abstract

A vanadium pentoxide (V_2_O_5_) thin film with thermal annealing as an ionic storage layer for electrochromic devices is presented in our study. The V_2_O_5_ thin film was deposited on an ITO glass substrate by an RF magnetron sputtering. The electrochromic properties of the film were evaluated after various thermal annealing temperatures. The structural analysis of the film was observed by X-ray diffraction (XRD), field emission electron microscopy (FE-SEM), and atomic force microscopy (AFM). The structure of the V_2_O_5_ thin film transformed from an amorphous to polycrystalline structure with directions of (110) and (020) after 400 °C thermal annealing. The electrochromic properties of the film improved compared with the unannealed V_2_O_5_ thin film. We obtained a charge capacity of 97.9 mC/cm^2^ with a transparent difference ΔT value of 31% and coloration efficiency of 6.3 cm^2^/C after 400 °C thermal annealing. The improvement was due to the polycrystalline orthorhombic structure formation of V_2_O_5_ film by the rearrangement of atoms from thermal energy. Its laminate structure facilitates Li^+^ ion intercalation and increases charge capacity and transparent difference.

## 1. Introduction

Electrochromic materials are important for their fascinating optical properties, such as reversible modulation of transmittance color and variation under a small voltage stimulus [1,2]. Electrochromic devices (ECDs) attract attention in various applications, such as electronic displays, smart windows, electronic papers, and information storage [3,4,5,6].

Typical ECDs have a sandwich structure with five superimposed layers either on a single substrate or positioned between two substrates as a laminate. The five layers include the electrochromic (EC) layer, the ions storage layer, the electrolyte, and two transparent conducting layers [7,8]. For the EC layer, the active cations (Li^+^, Na^+^, and H^+^) intercalate into the EC layer through a small bias voltage and deintercalation from the EC by a reverse bias voltage. This process bleaches/colors the EC film through the stimulus [9,10,11].

It is known that transition metal oxide thin films exhibit electrochromism. The main representatives of these oxides are tungsten oxide (WO_3_) [12,13], molybdenum oxide (MoO_3_) [14], vanadium oxide (V_2_O_5_) [15,16], and titanium oxide (TiO_2_) [17,18]. Various research groups have studied these oxides in the past decade since Deb’s discovery of the electrochromic phenomenon [19].

Tungsten oxide, WO_3_, is the most widely studied electrochromic material. It has high coloration efficiency, good stability, is less costly [12,20,21,22], and presents a characteristic dark blue color when absorbed by the near IR. Vanadium pentoxide, V_2_O_5_, on the other hand, has also drawn scientific attention as an electrochromic counter electrode (ionic storage layer) [23,24,25] because of its good electrochemical properties. In addition, it exhibits both anodic and cathodic electrochromism, which is useful for electrochromic devices. V_2_O_5_ thin film shows electrochromism with its reversible color changes between yellow and gray when an external stimulus voltage is applied [26] because its stacked laminate structure facilitates ion intercalation [27]. However, the electrochromic properties of a pristine V_2_O_5_ include low charge capacity and low transparent difference between colored and bleached states [28,29,30]. To overcome these problems, some studies have addressed the synthesis and fabrication of nanostructured V_2_O_5_ film to improve the film’s electrochromic properties [31]. Examples of techniques used to deposit electrochromic oxide films include Margoni et al. [32], who deposited V_2_O_5_ films by the hydrothermal method and thermal annealing. The film was prepared at 230 °C for 4 h and annealed at 500 °C for 1 h, showing a relatively high redox peak current density and diffusion coefficient than other films’ values. Panagopoulou et al. [6] reported V_2_O_5_ thin film deposition by RF-sputtering as a function of O_2_ content and substrate temperatures. Increased O_2_ content increases the grain size of the films, whereas the increase in substrate temperature leads to platelets perpendicular to the substrate, enhancing the films’ porosity. Zhu et al. [33] presented V_2_O_5_ micro/nanorods through heat treatment for electrospun composite fibers. Their results showed a high lithium storage performance, possibly due to how micro-/nano-structure samples disperse. They have high structural stability and efficient electron/ion transportation in the charging and discharging processes. Iida et al. [34] prepared V_2_O_5_ thin films using a pulsed-laser deposition technique. They demonstrated that the structure of the V_2_O_5_ thin film deposited at a low substrate temperature was broken by the insertion and extractions of lithium ions with an increasing cycle number of electrochemical reactions. Sputtering is an efficient technique for the deposition of thin films and can successfully deposit mixed oxides while maintaining control of sputtering power toward targets, gas flow rate, deposition thickness, and substrate heating temperature.

In order to obtain a better quality V_2_O_5_ thin film in the application of an ionic storage layer of ECs, the V_2_O_5_ thin film was deposited through an RF sputtering system. Then, it was annealed in a furnace to improve its electrochromic properties, including charge capacity, transparent difference, and coloration efficiency of the V_2_O_5_ film for ECs application. Our results showed the effectiveness of furnace annealing toward V_2_O_5_ thin film. The charge capacity of 97.9 mC/cm^2^ with a transparent difference ΔT value of 31% and coloration efficiency of 6.3 cm^2^/C was obtained from the V_2_O_5_ film after 400 °C thermal annealing as an EC ionic storage layer. The improvement was due to the formation of a polycrystalline orthorhombic structure in V_2_O_5_ film, whose laminate structure promotes Li^+^ ion intercalation and increases charge capacity. 

## 2. Experiments

The V_2_O_5_ thin film was deposited onto an ITO glass substrate with a resistivity of 6 Ω-cm for structural analysis and EC application. The cleaning process these glass substrates included acetone, isopropanol, and DI water for 5 min in sequence to remove surface contamination. Then, they were dried with pure nitrogen before being placed into the deposition chamber. The dimensions of these glass substrates were 4 × 2 cm^2^. An RF co-sputtering system was applied to deposit thin film. A V_2_O_5_ ceramic target with a purity of 99.9% and diameter of 7.62 cm was selected as the deposition source of the film. Pure argon with a purity of 99.99% was used as the sputtering gas and oxygen. The same purity was also inlet to the chamber considering the deposition with oxygen partial pressure. The base pressure of the chamber was pumped down to 5 × 10^−5^ Torr by a turbo pump. The flow rate of the inlet argon was 5 sccm, and the working pressure was kept constant at 5 mTorr. The ITO substrate did not have any additional heating during deposition. For the deposition of V_2_O_5_ thin film, the RF power directed toward the V_2_O_5_ target was 120 W and the duration of deposition time was 3 h. For the evaluation of the thermal annealing effect, the film was annealed in a furnace in the air environment at 200, 300, and 400 °C for 1 h, respectively. The thickness of the V_2_O_5_ thin film had an average value of 288.5 nm after deposition as measured by a profilometer (Dektak 6M stylus profilometer). It showed little change in thickness after thermal annealing.

We evaluated the structural analysis of the annealed samples using various analysis tools. An X-ray diffraction analysis (XRD) was performed on a Rigaku 18 KW Rotating Anode X-ray Generator with a continuous scan of Cu Kα radiation at λ = 1.5418 Å. The surface morphology was examined by a field-emission scanning electron microscope (FE-SEM, JEOL JSM 7000F). The topographical properties of the films were characterized by atomic force microscopy (AFM, Seiko HV-300). The optical properties of the film were recorded by a UV-Vis (Hitachi Ultraviolet-Visible 2008A Spectrophotometer) in the wavelength range of 300–1100 nm with a scanning rate of 400 nm/min to show the difference in the film’s transparency between colored and bleached states. The transmittance of the colored state was examined as the V_2_O_5_ film was biased at −2.5 V and the bleached state was biased at 2.5 V.

We used a Cyclic voltammetry (CV) method to measure the electrochromic properties of the films. The CV measurement system was performed using a potentiometer connected to a three-electrode cell. The three-electrode cell consisted of V_2_O_5_ film with a working electrode, a silver wire as a reference electrode, and a platinum foil as a counter electrode. The electrolyte in this measurement was a 0.1 M lithium perchlorate (LiClO_4_). The CV measurements were performed in a water-free environment. The CV measurements were evaluated at a potential range from −2.5 to +2.5 V with a speed of 10 mV/s. 

The charge density (*Q*) of the films was evaluated by the CV curve from the following relation [35]:

(1) $$Q\left(charge\;density\right)=\frac{1}{\nu \cdot A}\int_{V_{i}}^{V_{f}}I\left(V\right)dV$$

where *ν* is the sweep rate; *V_i_* is the initial sweep voltage; *V_f_* is the final sweep voltage; *A* is the area of the sample; and *I* is the current. The coloration efficiency (*CE*) is defined as the change in optical density at a specific wavelength divided by the inserted charge density (*Q*), as expressed in the following equation:

(2) $$CE\left(\lambda \right)=\frac{ln\left(\frac{T_{bleach}}{T_{color}}\right)}{Q}\;({\mathrm{cm}}^{2}/C)$$

where, *T_bleach_* is the transmittance of the film in the bleached state; *T_color_* is the transmittance of the film in the colored state; Transmittance is at the wavelength of 650 nm; and *Q* is the insert charge density. High coloration efficiency provides a large optical modulation with low charge insertion or extraction and is a crucial parameter for practical electrochromic devices. 

## 3. Results

**Figure 1:**
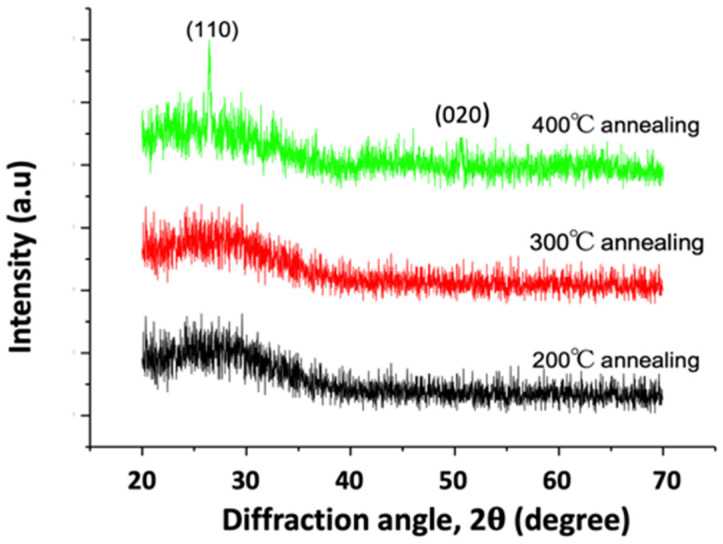
The XRD patterns of V_2_O_5_ thin film after thermal annealing at 200, 300, and 400 °C, respectively.

Figure 1 shows the XRD spectrum of the V_2_O_5_ thin film after thermal annealing at 200, 300, and 400 °C, respectively. The spectrum of the 200 and 300 °C-annealed samples is an amorphous structure. The XRD analysis did not detect any diffraction peak. As the annealed temperature increased to 400 °C, two diffraction peaks were detected in the V_2_O_5_ thin film. Two reflection peaks were observed at 2θ values of 26.3° and 50.5°, corresponding to (110) and (020) reflections of orthorhombic structure. All XRD peaks matched the orthorhombic phase of V_2_O_5_ (JCPDS card No. 00-041-1426) and were similar to the results reported by Abd-Alghafour et al. [36] and Qin et al. [37]. This finding reveals that the V_2_O_5_ thin film had a new phase of polycrystalline growth after the 400 °C-thermal annealing. 

**Figure 2:**
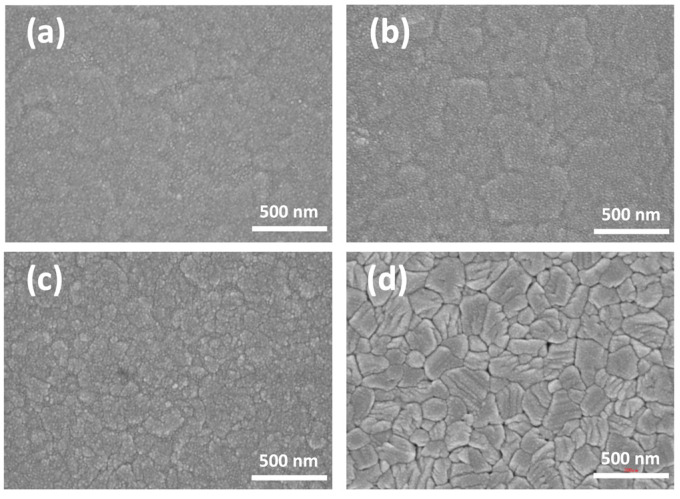
The surface morphology of V_2_O_5_ thin film after thermal annealing at various temperatures with a magnification of 50,000× (**a**) without annealing (**b**) 200 °C, (**c**) 300 °C, and (**d**) 400 °C, respectively.

The FE-SEM observed the surface morphology of V_2_O_5_ thin films after thermal annealing at various temperatures at a 50,000× magnification, as shown in Figure 2a–d. The unannealed V_2_O_5_ sample shows a smooth surface morphology. The 200 °C-thermal annealed V_2_O_5_ thin film had a flat island shape and a dense surface without cracks. The sample was annealed in the furnace with the atmospheric environment, while the oxygen in the environment filled the film defects and led to a smooth surface morphology. As the annealing temperature increased to 300 °C, small aggregations formed on the film, and the surface morphology became rougher than the 200 °C-annealed sample. As the annealing temperature increased to 400 °C, an increase in the order of polycrystalline in the V_2_O_5_ film was revealed by the XRD analysis. This thermal annealing showed considerable effects on the surface morphology with different sizes (about 50~200 nm) of grains, as shown in Figure 2d. The voids and cracks on the images are the grain boundaries of various grain sizes. Higher annealing temperatures provide higher energy to transform the V_2_O_5_ film from an amorphous to polycrystalline structure with various grain sizes.

**Figure 3:**
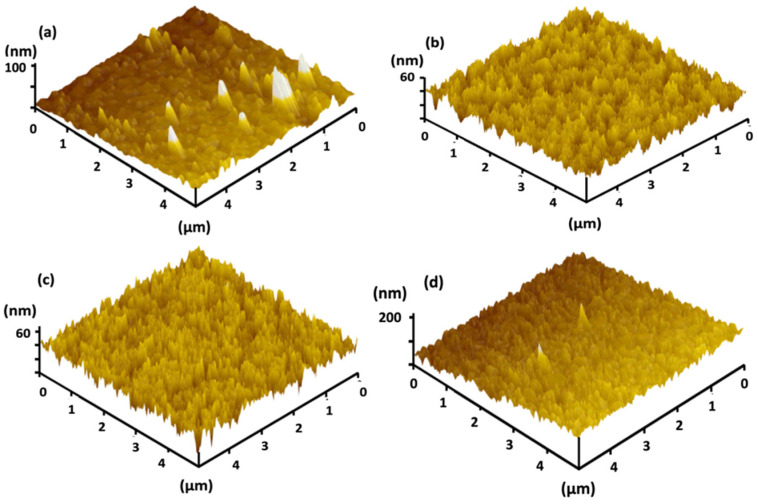
Three-dimensional AFM images of V_2_O_5_ thin films (**a**) without annealing (**b**) 200 °C-annealing, (**c**) 300 °C-annealing, and (**d**) 400 °C-annealing.

Figure 3 shows the AFM morphological and topographic characteristics of the V_2_O_5_ prepared with various thermal annealing temperatures, measured over 5 μm × 5 μm areas. The RMS (root mean square) roughness of the unannealed sample is 3.45 nm, and that of the V_2_O_5_ film slightly increased to 5.23 and 5.87 nm after thermal annealing at 200 and 300 °C, respectively. A similar value of 5.9 nm was also obtained for V_2_O_5_ thin films produced using the sol-gel and dip-coating methods, deposited onto ITO substrates, and heat-treated in the air at 300 °C for 12 h [38]. As the annealing temperature increased to 400 °C, the RMS roughness increased to 27.22 nm, which was attributed to the polycrystalline structure of the film.

**Figure 4:**
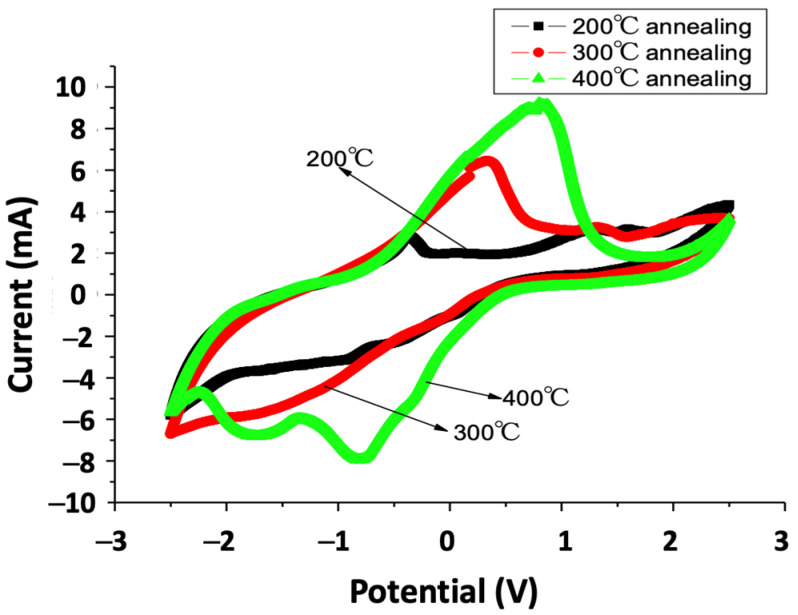
Cyclic voltammetry of the thermal annealed V_2_O_5_ thin film.

We used CV measurement to investigate the effectiveness of V_2_O_5_ thin film as an ion storage layer for electrochromic devices; the obtained results are displayed in Figure 4. The CV curve of pure V_2_O_5_ thin film without thermal annealing shows a cathodic reduction peak (−1.6 V) attributable to Li^+^ intercalation and an anodic oxidation peak (−0.4 V) that corresponds to Li^+^ extraction [28]. The CV measurement of the unannealed V_2_O_5_ thin film had a charge capacity of 54.7 mC/cm^2^, as revealed in our previous work [39]. The voltammogram of 200 °C-annealed V_2_O_5_ thin film (black line) shows an oxidation peak at the potential of −0.4 V and a slight reduction peak near the potential of −1 V. As the annealing temperature increased to 300 °C, the oxidation peak became strong, and the peak moved to the potential of 0.4 V; the reduction process showed Li^+^ ions intercalated into the V_2_O_5_ film as compared to the 200 °C-annealed sample. As the annealed temperature increased to 400 °C, the oxidation peak’s potential increased to 1 V. There were two reduction peaks between 0 and −2 V shown in the cathodic potential. These peaks revealed more intercalation of Li+ ions during the reduction process. The ionic storage capacity of the V_2_O_5_ thin film increased with the annealing temperature. The charge capacity increased from 54.7 mC/cm^2^ of the unannealed sample to 69.7 and 97.9 mC/cm^2^ of the 300 and 400 °C-annealed samples, respectively. Compared to the literature on V_2_O_5_ thin film, Ottaviano et al. [24] studied the dependence of the electrochromic properties of sputtered V_2_O_5_ films on the oxygen flow used during deposition and reached values of 49.8 mC/cm^2^. Panagopoulou et al. [40] measured charge density for intercalation/de-intercalation of 37.55 mC/cm^2^ and transmittance modulation of 9.1 and 12.2 at the wavelengths of 560 and 750 nm, respectively. Panagopoulou et al. [6] presented enhanced charge storage properties of 553 mAh/g and transmittance modulation of 30.4, 27.5, and 18.6 at the wavelengths of 400, 560, and 750 nm, respectively. The increased amounts of charge capacity promote the thermal annealing effect of V_2_O_5_ thin film as an ionic storage layer for electrochromic devices.

**Figure 5:**
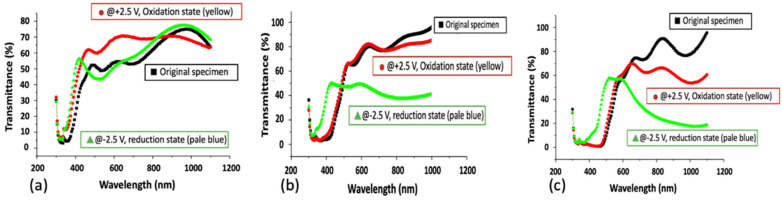
The transmittance of the thermal annealed V_2_O_5_ thin film, the original sample (square), oxidation state (circle), and reduction state (triangle), after annealing at (**a**) 200, (**b**) 300, and (**c**) 400 °C, respectively.

The effectiveness of thermal annealing on the optical properties of V_2_O_5_ film was evaluated in the wavelength range from 300 to 1100 nm for the 200 °C-annealed and 400 °C-annealed samples and in the wavelength range from 300 to 1000 nm for the 300 °C-annealed sample. The results are shown in Figure 5a–c. The three curves in Figure 5 include the original specimen (without bias), oxidation state (bias at +2.5 V), and reduction state (bias at −2.5 V). They are shown in order of the curves as a black square, red circle, and green triangle. The transmittance difference is defined as ∆T = T_bleach_ − T_color_ at the incident wavelength of 650 nm. Table 1 is the summary of the transmittance difference, charge capacity, and coloration efficiency of the V_2_O_5_ thin film after various annealing temperatures. Table 2 shows the photographs of the original, bleached, and colored states of the V_2_O_5_ thin films without and after various thermal annealing temperatures. The ∆T value at the 650-nm wavelength of the unannealed V_2_O_5_ thin film is 15%, as revealed in our previous work [39]. As it was annealed, the transmittance difference increased to 31% for the 400 °C-annealed sample due to the grain growth of the film’s polycrystalline structure created by the rearrangement of atoms through thermal energy. The crystalline laminated structure facilitates the intercalation of Li^+^ ions and promotes the charge storage capacity. These effects lead to increased transmittance differences between bleached and colored states. Photographs of the original, bleached, and colored V_2_O_5_ films are shown in Table 2. It is known that the color of bleached V_2_O_5_ film is yellow, and the colored V_2_O_5_ film is gray [6]. The unannealed, 200, and 300 °C-annealed samples showed similar results in our study. The photograph of the colored 400 °C-annealed V_2_O_5_ thin film showed a dark green color. For the 400 °C-annealed V_2_O_5_ thin film, an increase in the order of polycrystalline structure was detected by the XRD analysis. The transmittance of the 400 °C-annealed sample had an overlaid spectrum of the original and oxidation (bleached) state in the wavelength between 400 and 700 nm, as shown in Figure 5c. Moreover, the reduced (colored) state of the 400 °C-annealed sample had a blue shift at the absorption edge of the transmittance spectrum. S.F. Cogan et al. [39] reported that in response to lithium intercalation, the fundamental optical absorption edge of V_2_O_5_ shifts to higher energies by 0.20–0.31 eV as the lithium concentration increases from Li_0_._0_V_2_O_5_ to Li_0_._86_V_2_O_5_. The absorption edge shift significantly decreases in absorbance from the 350 to 450 nm wavelength range. The transmittance difference between bleached and colored states in the 400 °C-annealed sample at a wavelength of 600 nm is very small (5.4%), whereas it shows a larger transmittance difference between bleached and colored states at 31% with a wavelength of 650 nm, as shown in the spectrum of Figure 5c. The transmittance spectrum of the reduction state process shows the absorption of blue (about 400~500 nm), red (about 620–750 nm), and higher transparency of green (about 490~580 nm). Therefore, the photograph of the colored state for the 400 °C-annealed V_2_O_5_ thin film is green.

The coloration efficiencies of V_2_O_5_ thin films after various thermal annealing temperatures are summarized in Table 1. The coloration efficiency (CE) of the V_2_O_5_ thin film is 3.6, 3.9, 8.9, and 6.3 cm^2^/C for the sample without annealing, and 200, 300, and 400 °C for thermal annealing, respectively. The CE improvement in the annealed samples (e.g., 300 and 400 °C annealing) is due to the increasing order of polycrystalline structure by the rearrangement of atoms through thermal energy. The high coloration efficiency value of the electrochromic film means that electrochromic devices should have adequate optical transmittance modulation with less intercalation of charge, causing better stability and reproducibility of colored/bleached cycles. The 300 °C-annealed sample had a higher transmittance modulation and lower intercalation of charge than the 400 °C-annealed sample. Therefore, it had a higher coloration efficiency.

## 4. Conclusions

Thermal annealing effects on V_2_O_5_ thin film, which was deposited by RF sputtering as an ionic storage layer of the electrochromic device, are presented. The structure of pristine V_2_O_5_ film is amorphous and transferred to a polycrystalline structure after 400 °C thermal annealing. The polycrystalline laminated structure of the V_2_O_5_ thin film promoted the intercalation of Li^+^ ions and increased charge storage capacity. The charge capacity of the 400 °C-annealed V_2_O_5_ film as an ionic storage layer increased from 54.7 to 97.9 mC/cm^2^ compared with an unannealed sample. The incompletion of Li^+^ ion de-intercalation from the V_2_O_5_ laminated structure at the oxidation (bleach) state of the 400 °C-annealed sample led to a lower transmittance. Therefore, the transmittance difference of the 400 °C-annealed sample decreased slightly compared with the 300 °C-annealed sample.

## Figures and Tables

**Table 1:** Summary of the transmittance difference, charge capacity, and coloration efficiency of the V_2_O_5_ thin film after various temperature annealing.

Annealing Temperature	Without	200 °C	300 °C	400 °C
**∆T** **(%) at 650 nm**	15%	13%	37%	31%
**Charge capacity (mC/cm^2^)**	54.7	52.6	69.7	97.9
**Coloration efficiency (cm^2^/C)**	3.6	3.9	8.9	6.3

**Table 2:** The photographs of colored and bleached states of the V_2_O_5_ thin films after various temperature annealing.

Annealing Temperature	Without	200 °C	300 °C	400 °C
**Original**	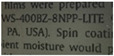	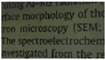	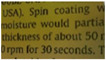	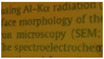
**Bleached**	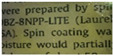	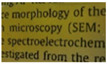	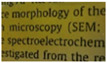	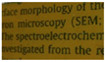
**Colored**	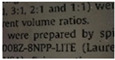	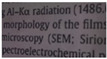	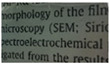	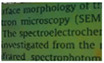

## Data Availability

Not applicable.
